# The Use of Isolated Human Lymphocytes in Mycotoxin Cytotoxicity Testing

**DOI:** 10.3390/ijms9081515

**Published:** 2008-08-25

**Authors:** Pholo W. Maenetje, Neil de Villiers, Mike F. Dutton

**Affiliations:** Food, Environment & Health Research Group, University of Johannesburg, P.O. Box 17011, Doornfontein 2028, South Africa

**Keywords:** Cytotoxicity, mycotoxins, mycotoxin screening, lymphocytes, mycotoxicosis

## Abstract

The cytotoxicity of selected mycotoxins against isolated human lymphocytes was investigated, as a means of detecting mycotoxins in extracts derived from cereal samples. The methodology was based on the ability of viable cells to reduce methyl tetrazolium bromide to a purple formazan dye that could be quantitated by spectrophometric means and hence give a measure of the cytotoxicity of added substances. The results showed that there was good correlation with the occurrence of identified mycotoxins with only a minimum of false positives. For example, of the 13 samples of barley or barley derivatives that were positive for the mycotoxins, fumonisin B_1_ (FB1) deoxynivalenol (DON) and ochratoxin A (OTA), all gave positive cytotoxicity responses. Two samples negative for mycotoxins gave no cytotoxicity responses. There was little variation between the results for lymphocytes drawn from the same healthy volunteer on three different occasions. Furthermore, for two of the mycotoxins tested (FB1 and DON) it was possible to correlate general levels of mycotoxins present to the cytotoxic response of the lymphocytes but not for OTA, where it was concluded that interfering substances prevented direct correlation. It was concluded that this method was suited for general application as it could handle relatively high number of samples in a short period of time.

## 1. Introduction

Since mycotoxins are fungal secondary metabolites [1], they are typically not chemically similar to essential metabolites found in cells, and form a collection of organic molecules that have unique and individual structures. This means that there is no unified way of analysing them with conventional instrumentation and usually each one has to be individually quantitated, which is tedious and expensive. The situation is exacerbated by the fact that over 300 such toxins are known [2] and it is likely that there are others as of yet unknown. Commercially only about six of these are regarded as being significant [3], but in rural subsistence crop production and storage, the number of mycotoxins found may be larger, not the least because of the poor agricultural situation in these areas, lack of proper storage facilities and quality control. A major problem with regards to the occurrence of mycotoxins in agricultural commodities is the possibility that they will end up in animal feed with a subsequent loss in animal production or even death [4]. This problem is further compounded by the carryover of these toxins into the food chain, particularly through secretion into milk and thence dairy products [5]. Mycotoxins are, therefore, of considerable importance with regards to animal health and commercial production and the entry of mycotoxins into the human food chain. There are several approaches that can be taken to screen for dangerous levels of toxicity in crops and feed but these are often expensive, have limits on the number of toxins identified and are not one hundred percent reliable. This not only makes preventative action difficult to apply, but also means that retrospective studies on food contaminants are not accurate, where dangerous toxins are missed or new ones not identified.

Several approaches may be taken to remedy this, e.g., by doing a fungal screen, which can give some indication of toxins that are likely to be found based on our knowledge about the mycotoxins produced by the detected fungal species. This method can only give an indication, as some fungal strains of potential toxin producing species do not produce the toxin, whereas other infecting species may have died out prior to screening but left their toxin in the matrix. Another approach, which is advocated in this presentation, is to screen extracts of agricultural commodities and food for cytotoxicity using human and other cell lines [7]. This methodology has the advantage of using target cells [8] whilst avoiding whole animal experiments. It has been used more recently to detect toxicity in environmental systems [9] including agricultural products [10]. The methodology can be developed to be fairly rapid (a result can be obtained after 12 hours incubation) cover most toxic substances and the cytotoxicity can be expressed quantitatively against an untreated control. Some disadvantages with the method are that it may be expensive, in that cell lines have to be grown and maintained in a specialised aseptic facility and the test can give rise to false positives. The question of expense and high technology facility is, important to laboratories in Africa. Consequently the cytotoxicity-testing approach has been modified to using cells available to laboratory workers, i.e., blood cells, in particular, lymphocytes. (Clearly before these can be used on a routine basis, ethical considerations will have to be met, including agreement between the parties involved and proper medical supervision will be required.) These do need to be handled under sterile conditions, which do require careful aseptic technique and a certain amount of expense but they do not need long maintenance under sterile conditions like cell cultures, as they need shorter maturation times.

The method used here for the testing of cell viability is based on that of Hanelt *et al*. [11] whereby grown or isolated human or animal cells are exposed to the toxin or potentially toxic extracts under test. The cells are incubated for at least 12 hours and their viability is measured against a control treated exactly as the same as the tests but without toxic principles, after adding a reagent (containing methyl thiazole tetrazolium salt (usually bromide) [MTT]) that is reduced to a formazan purple dye by the redox potential generated by the respiring living cell. The amount of dye that is released is proportional to the number of viable cells and this can be measured by a plate reader capable of quantitatively measuring light absorption. At least 3 experiments per concentration are set up to be tested at an increasing dose of toxin or extract, so as to obtain a progressive response. In this study the commodity examined was barley and barley malt but the method can be applied to most agricultural commodities.

## 2. Materials and Methods

### 2.1. Extraction of mycotoxins from barley samples

Samples of barley and malted barley were obtained from a South African maltster and retail outlets in Gauteng. The initial method of screening followed that devised by Patterson and Roberts [12] and briefly consisted of milling and thoroughly mixing the sample. Extraction of a 25 g sample was achieved using acetonitrile/aqueous potassium chloride (4%, 9:1 v/v, 100 mL) shaken for 1 hour and then filtered. The filtrate was extracted with dichloromethane (DCM) (three equal volumes) after addition of sodium bicarbonate solution (30 mL saturated solution diluted with 20 mL water) to obtain a “neutral” (N) fraction (containing DON and other non-acidic mycotoxins) and after re-acidification with three equal volumes of DCM to obtain and “acid” (A) fraction (containing OTA and other acidic mycotoxins). The N fraction was then subjected to dialysis against 30% (v/v) aqueous acetone over night and then back extracted into DCM. Both fractions were transferred to sealed vials as dried extracts, which were reconstituted in a small known volume of carrier solvent depending upon the further procedures, i.e., DCM for thin layer chromatography (TLC) and dimethylsulphoxide (DMSO) for cytotoxicity testing. Fumonisin B_1_ (FB1) extraction was done by using the clean up method of Sydenham *et al.* [14]. This involved extracting the ground barley with methanol/water (3:1) and then passing the extract through a strong anion cartridge (SAX). The absorbed fumonisin was then eluded with 1% acetic acid in methanol after washing.

### 2.2. Cytotoxicity test

Blood (12 mL) was obtained from three healthy donors into heparinised tubes and mixed with an equal volume of culture medium (RPMI-1640, Highveld Biologicals, South Africa) supplemented with 10% foetal calf serum) and overlaid on Histopaque 1077 (Sigma) and centrifuged at 800 xg for 30 min. The interface layer consisting of mononuclear cells were removed using a sterile pipette. The isolated lymphocytes were washed three times with CCM (5 mL) with centrifugation for 10 min after each wash (adapted from Boyum (1968) [13] (ethical clearance, Ethic Committee 2003, Faculty of Health Sciences, Technikon Witwatersrand). The washed pelleted cells were then resuspended in CCM (10 mL) containing phytohaemagglutinin-p (1%, Sigma Ltd.) and cultured in 96-well flat bottomed tissue plates (each well containing 100 μL cell suspension, having 10^6^ cells/mL plus 100 μL test solution) and incubated at 37 °C in a 5% CO_2_-buffered and humidified incubator for 3 days. For cytotoxicity assays, triplicate wells of cultured cells were treated with pure mycotoxins and barley fractions at concentrations ranging from 3,125 to 50,000 ppb of fumonisin B_1_ (FB1) (main mycotoxin contaminant of maize) or deoxynivalenol (DON) (main mycotoxin contaminant of barley) and neutral mycotoxin extracts from barley (DON estimated from analysis) and 31.2 to 50 ppb ochratoxin A (OTA) (also a major contaminant of barley) and acid mycotoxin fractions from barley, together with suitable controls. Controls consisted of cells treated with the carrier solvent at levels consistent with those used to add mycotoxin to the culture and with extracts that had been shown by chromatography not to contain common mycotoxins. These cell cultures were then further incubated for 24 hrs in a 37°C in a carbon dioxide (5%)-buffered and humidified incubator. Exposed cell cultures were then evaluated by the MTT assay [13] with minor modifications. The medium was removed using a multi-channel pipette and DMSO was added to solubilise the released purple formazan dye. This was measure at 510 nm on an Elisa plate reader. Results were plotted, readings versus amount of extract or toxin and compared to those given by the control and expresses as a percentage cell viability of the control. Data obtained from the cytotoxicity assay was analysed by the t-test using Sigmastat 3.10. Mean values were deemed to be significantly different if the level of probability was ≤0.05.

### 2.3. Analysis of the extracts

The basic screening was done by two dimensional thin layer chromatography (TLC) on silica gel G aluminium backed plates with added fluorescent indicator (Merck Art 5554) cut to 10 cm squares, which allowed for the detection of the most commonly occurring mycotoxins [12]. Mycotoxins were detected by inspection under long wave UV light or by spraying with suitable visualising agents. Semi-quantification was done by visual comparison of detected mycotoxins against known standards. Further more definitive analysis was done using the Vicam immunoaffinity/fluorimetry method (Vicam Ltd, Waterton, USA) and/or gas chromatography/mass spectrometry for DON after derivatization [15].

## 3. Results and Discussion

### 3.1. Effect of known mycotoxins and barley extracts on lymphocytes

**Figure 1:**
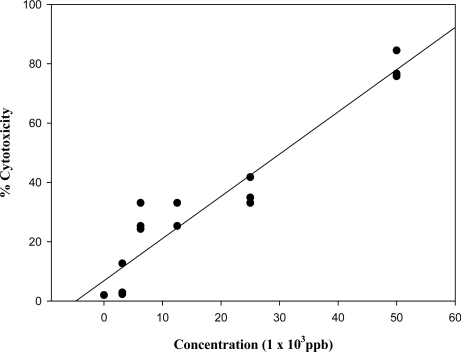
Dose- response linear regression curve of fumonisin B_1_ standard on the human lymphocyte cell line (r = 0.958). All plotted dots of the curves represent means of 3 measurements in 3 distinct test procedures.

**Figure 2:**
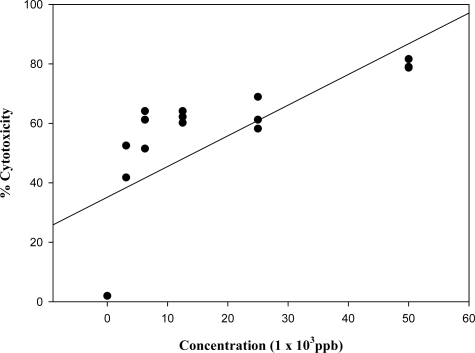
Dose-response linear regression curve of deoxynivalenol on the human lymphocyte cell line (r = 0.713). All plotted dots of the curves represent means of 3 measurements in 3 distinct test procedures.

**Figure 3:**
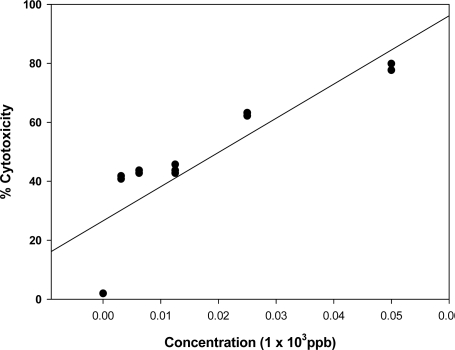
Dose- response linear regression curve of ochratoxin A standard on the human lymphocyte cell line (r = 0.819). All plotted dots of the curves represent means of 3 measurements in 3 distinct test procedures.

All mycotoxin standards evaluated in this study were toxic to the human lymphocytes (Figures 1 to 3). The toxicity of FB_1_ on the lymphocytes is presented by the dose-response regression curve (Figure 1) after 24 hr of incubation. The regression coefficient of FB_1_ on the lymphocytes was found to be 0.958 with an IC_50_ (concentration of the tested inhibitor resulting in a 50% reduction of viable cells as measured by MTT assay of 29,160 ppb, Figure 1). There was no statistical difference in the sensitivity of lymphocytes to FB_1_ among the three donors. Interestingly a slight increase in cell viability at a lower concentration of 3,130 ppb FB_1_ was observed, which is in contrast to other studies using other cell lines [16] however, a decrease in cell viability was observed at higher concentrations ranging from 6,250 to 50,000 ppb FB_1_. Concentrations ranging between 6,250 and 25,000 ppb showed no significant difference with cell viability ranging between 80 and 60% but concentrations of 50,000 ppb FB_1_ resulted in a significant reduction in cell viability (≥ 42%) relative to 25,000 ppb FB_1_ treatment (p<0.05). The proposed mechanism of FB1’s toxicity is its inhibition of the enzyme sphingosine *N*-acetyltranferase [17] which results in an imbalance in the sphingoid base level in the cell and leads to lipid-mediated alterations in the signalling of metabolic pathways. This in turn may affect cell proliferation, apoptosis, differentiation, morphology [18] and in extreme cases of human exposure could possibly lead to oesophageal cancer [19].

Deoxynivalenol was found to be more toxic to the lymphocytes (IC_50_ = 5,500 ppb) compared to FB_1_ after 24 hr of incubation (p<0.05) (Figure 2). However, a similar trend to DON in toxicity response was observed, as there was no significant difference in cell viability at concentrations ranging between 3,125 and 25,000 ppb. Concentrations of 50,000 ppb also showed a significant reduction in cell viability (20%) as compared to 25,000 ppb DON treatment (p<0.05).

The effects of DON in this study agree with others, where it has been shown to be a potent inhibitor of lymphocyte proliferation, which could in turn reflect on its ability to inhibit protein synthesis by binding to ribosomal peptidyl transferase [20, 21]. These effects have been also shown to alter the humeral immunity and cell-mediated immunity [22] or immuno-stimulatory effects depending upon the dose and duration of exposure [22] thereby resulting in increased negative action.

Deoxynivalenol and FB_1_ were found to be less cytotoxic (p<0.05) than OTA (Figure. 3, IC_50_ = 20 ppb (20 ppb OTA) after 24 hrs of incubation. The regression coefficient of OTA toxicity to the lymphocytes was found to be 820 ppb. Once again, a similar trend was also observed, as there was no significant difference in cell viability at low concentrations less than 25 ppb OTA on the lymphocytes. Contrary to the effects of DON and FB_1_, no dose dependant reduction in cell viability (≤ 17 %) was observed at a higher concentration of 50 ppb OTA with respect to 25 ppb OTA treatment.

The variation of the individual’s lymphocytes sensitivity to the tested mycotoxins in this study (DON, FB_1_ and OTA) was found to be negligible, as no significant difference was observed among the three donors. However, potential differences in *in vivo* exposures of lymphocytes to toxins must take into account individual differences such as the uptake, metabolism, combined effects of the toxins, the extent of exposure (duration or dose) and other factors such as age, gender, nutritional status, as well as the physiological state of the affected individuals.

**Figure 4:**
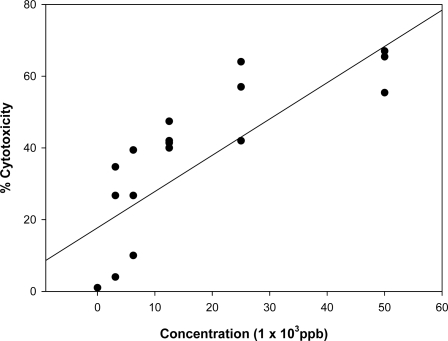
Dose- response linear regression curve of neutral barley fraction (B4) on the human lymphocyte cell line (r = 0.811). All plotted dots of the curves represent means of 2 measurements in 3 distinct test procedures.

**Figure 5:**
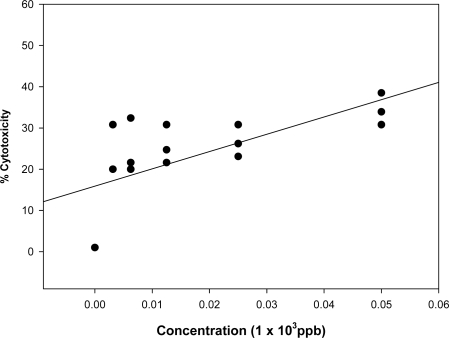
Dose- response linear regression curve of acid barley fraction (B4 Concentration = ochratoxin A equivalent from instrumental analysis)) on the human lymphocyte cell line (r = 0.714). All plotted dots on the curve represent means of 3 measurements in 3 distinct test procedures.

Examples of some of the dose response linear regression curves obtained from the neutral and acid and neutral barley extract fractions are presented in Figure 4 and Figure 5. The barley (sample B4) was found to contain 4,500 ppb DON by the Vicam method and the neutral fraction was adjusted to give the DON equivalent concentrations in the cytotoxicity test as presented in Figure 4. A comparison of the results in this Figure with those for the toxin alone in Figure 2, show a very similar dose response. The toxicity of the positive fractions for OTA (A fractions) on the human lymphocytes was also evaluated and an example of an acid barley fraction (B4 A) is given in Figure 5. A slight decrease in cell viability relative to the control (untreated cells) was observed with increased concentrations. The acid fractions were less cytotoxic (p<0.05) compared to pure OTA, with all concentrations in the low range (≤50 ppb), whereby most of the acid fractions exhibited cell viability above 60% and thus, IC_50_ values of the acid fractions were determined at concentrations greater than 50 ppb of OTA equivalent. Similarly to the pure OTA, no significant difference was observed in the cytotoxicity of the acid fractions at concentrations <50 ppb.

Cytotoxicity tests done on lymphocytes using the neutral fractions (7 extracts) showed similar toxicity compared to the mycotoxin standards. Although there was a correlation between neutral fractions and the DON and FB1 standards in terms of toxicity, contrarily TLC indicated low intensities of these mycotoxins in the fractions. In addition, GC-MS also detected low DON concentrations (63–832 ppb) in the neutral fractions. The Vicam method of analysis was used as the definitive method for FB1 and DON and the concentrations found by this method together with an assessment of the cytotoxicity of these extracts is given in Table 1. In general, the fractions exhibited toxic effects that were not solely explained by the contents of the detected mycotoxins by TLC or GC-MS, indicating that other mycotoxins or a combination of mycotoxins in the fractions even other toxic substances may have contributed to the toxic effects, comparable to the findings obtained by Bunger *et al.* [23].

The acid fractions (6 extracts), however, were found to be less cytotoxic compared to the OTA standard (p <0.05) resulting in a cell decrease below 40%. Low cytotoxicity of the acid fractions on the lymphocytes could have resulted from other interfering compounds in the fractions. In addition to TLC analysis of the acid fractions indicated low intensities (presence) of OTA in the acid fractions and thus correlates with the toxicity exhibited by these acid fractions on the lymphocytes.

### 3.3. Estimation of toxin concentration in barley fractions using the cytotoxicity test

The actual concentrations of FB_1_ in the barley samples determined from the Vicam quantitative analysis (Table 1) were found to correlate well with their estimated concentrations by cytotoxic response of the extracts (N) (r = 0.946) (Figure 6A). Similarly determined concentrations of DON in the barley extracts (N) (Table 1) also correlated well with the cytotoxic response (r = 0.801) (Figure 6B). Although there are other metabolites or mycotoxins in the barley fractions, the correlation of actual and estimated values for DON and FB_1_ suggests that the toxicity on the lymphocytes resulted primarily from these particular toxins.

As opposed to the correlation between analysis and cytotoxic response found for DON and FB_1_, none was found in the case of OTA positive barley extracts (A) (r = 0.371) (Figure 6C). The actual and estimated linear regression curve of OTA indicates two sets of data, one having high concentrations of OTA, as determine by analysis but resulting in low cytotoxicity and the other data indicating high cytotoxicity of extracts with low analytical concentrations.

These results show that the cytotoxicity caused by the OTA-containing fractions could be not explained by the absence or presence of OTA alone. The two sets of data obtained from the linear regression curve are probably due to the low levels of OTA in the fractions and/or other interfering compounds in the fractions, such as breakdown products of OTA or other mycotoxins. The results suggest that this technique may not be as effective in determining the amount of toxin present at lower levels (i.e., ppb levels) even though these are of more potent toxins such as OTA. However, all the 13 extracts that were positive for mycotoxins by the other analytical techniques were found to be positive by cytotoxicity assay and the four control extracts that were negative by analysis for mycotoxins were also negative by cytotoxicity testing.

## 4. Conclusion

The MTT assay used for the analysis of barley fractions on lymphocytes allowed pre-analytical screening to indicate, which barley samples had residual toxicity. The toxicity of pure mycotoxins on human lymphocytes was observed and compared to that of barley fractions. The MTT assay used allowed adequate comparability of the results among different mycotoxins, although it gave no further insights into the different modes of biochemical actions of the tested mycotoxins. In general the result were highly encouraging in that they showed that the cytotoxicity testing method was capable of detecting mycotoxins in cereal extracts and that for two toxins FB1 and DON there was a reasonable repeatable correlation between the response to known levels of mycotoxins standards and mycotoxins in extracts contained from barley samples. This would allow personnel in laboratories using this method to detect which extracts were deemed to need further analytical attention and in certain cases could predict the general level of toxin present. Hence the use of lymphocytes as a screening tool for agricultural commodity quality control can be further developed.

## Figures and Tables

**Figure 6A:**
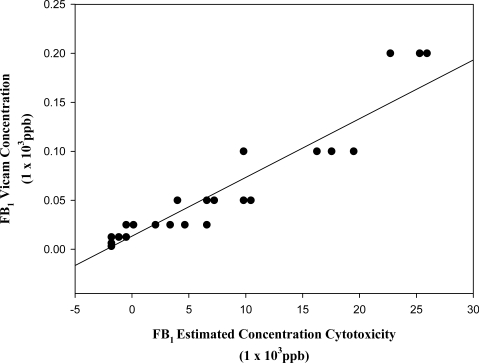
Linear regression curve of fumonisin B_1_ actual and estimated concentration values (r = 0.946).

**Figure 6B:**
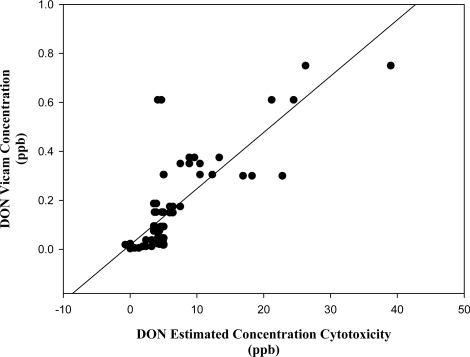
Linear regression curve of deoxynivalenol, actual and estimated concentration values (r = 0.802).

**Figure 6C:**
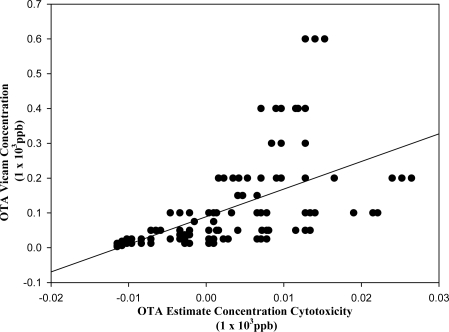
Linear regression curve of Ochratoxin, actual and estimated concentration values (r = 0.371).

**Table 1:** Summary of the cytotoxicity of the various barley fractions tested and their mycotoxin content by the Vicam method.

Sample	Cytotoxicity* ppb	FB1 ppb	DON ppb	OTA ppb
B4 N fraction	25,000	n.d.	4,800	−
B4 A fraction	>50	−	−	4
B7 A fraction	>50	−	−	1
B9 A fraction	50	−	−	2
B10 N fraction	6,250	n.d.	n.d.	−
B10 A fraction	>50	−	−	6
B11 N fraction				
FB1 fraction	50,000	40	900	−
B11 A fraction	>50	−	−	4
B14 N & A fractions	n.d.	n.d.	n.d.	n.d.
B21 N fraction	20,000	n.d.	7,500	−
FB1 fraction				
B30	18,000	n.d.	7,500	−
B31 N&A fractions	n.d.	n.d.	n.d.	n.d.
MB5 N&A fractions	>50,000	n.d.	n.d.	−
MB24				
N fraction	>50,000	71	6,200	−
FB1 fraction				
PB4 A fraction	20	−	−	2

* Cytotoxicity figure value at half the cytotoxicity level of the control, i.e. 50% of untreated lymphocytes; ppb = parts per billion (μg/kg) in the original sample. As 200 μL are derived from 25g of original sample and 20 μL are used in a total 200 μL of culture fluid per well the actual concentration experienced by the lymphocytes at the highest concentration will be: ¼,000 of this and the lower levels appropriate dilutions thereof; n.d. = not detected; - = not found in these fractions.

FB1 = Fumonisin B1; DON = Deoxynivalenol; OTA = Ochratoxin A; B = barley grain; MB = malted barley; PB = pearl barley.
